# First MRI application of an active breathing coordinator

**DOI:** 10.1088/0031-9155/60/4/1681

**Published:** 2015-01-29

**Authors:** E Kaza, R Symonds-Tayler, D J Collins, F McDonald, H A McNair, E Scurr, D-M Koh, M O Leach

**Affiliations:** 1 CR-UK Cancer Imaging Centre, Institute of Cancer Research London and Royal Marsden Hospital, London, UK; 2 The Royal Marsden NHS Foundation Trust, UK; 3 Department of Radiotherapy, Royal Marsden NHS Foundation Trust and Institute of Cancer Research, Sutton, UK; Evangelia.Kaza@icr.ac.uk

**Keywords:** ABC, MRI, radiotherapy, lung cancer

## Abstract

A commercial active breathing coordinator (ABC) device, employed to hold respiration at a specific level for a predefined duration, was successfully adapted for magnetic resonance imaging (MRI) use for the first time. Potential effects of the necessary modifications were assessed and taken into account. Automatic MR acquisition during ABC breath holding was achieved. The feasibility of MR-ABC thoracic and abdominal examinations together with the advantages of imaging in repeated ABC-controlled breath holds were demonstrated on healthy volunteers. Five lung cancer patients were imaged under MR-ABC, visually confirming the very good intra-session reproducibility of organ position in images acquired with the same patient positioning as used for computed tomography (CT). Using identical ABC settings, good MR-CT inter-modality registration was achieved. This demonstrates the value of ABC, since application of T1, T2 and diffusion weighted MR sequences provides a wider range of contrast mechanisms and additional diagnostic information compared to CT, thus improving radiotherapy treatment planning and assessment.

## 1. Introduction

Respiratory motion presents a challenge for thoracic and abdominal medical imaging and treatment. Computed tomography (CT) and magnetic resonance imaging (MRI) suffer from artefacts and blurring induced by breathing. Radiotherapy treatment margins need to be increased to take into account tissue displacements due to respiration. Many techniques have been developed for motion compensation in radiotherapy (Guckenberger *et al* 2012) and in MRI (van Heeswijk *et al* 2012). The techniques most frequently applied in MRI are acquisitions during operator-guided self-induced breath holding or during a selected respiratory phase tracked by a navigator in free breathing. Nevertheless, navigator triggering has offered no advantages over simple free breathing on the diffusion parameters of abdominal diffusion-weighted (DW) MRI (Jerome *et al* 2014). A critical radiotherapy requirement is that the tumour remains at the same position during irradiation and that as much as possible healthy tissue is spared. For regions affected by respiratory motion, this is usually achieved by treating patients during a specific inhalational phase. However, instructing patients to hold their breath during imaging or treatment depends on their compliance and provides no certainty about the true onset, duration and stability of a breath hold. Moreover, individuals may not be able to reproduce exactly the same level of inspiration or expiration when breath holds need to be repeated.

To address these issues, a first active breathing control (ABC) device to monitor and stop patient respiration at a preselected phase of the breathing cycle for a predefined duration was developed by Wong *et al* (1999). Their ABC application suggested improvements of the CT intra-fraction reproducibility of liver and lung immobilisation. Subsequently, a respiratory control apparatus consisting of a turbine flow meter and capable of enforcing patient breath-holds at preselected volumes of respiration by inflating a balloon valve was developed and made commercially available under the name Active Breathing Coordinator (ABC) (Elekta Oncology systems Ltd, Crawley, West Sussex, UK). The term ABC will refer from now on to the commercial device by Elekta. This is successfully used during lung radiotherapy to reduce treatment margins (Brock *et al* 2011), during liver radiotherapy to improve intra-fraction reproducibility of liver position (Eccles *et al* 2006) and during breast irradiation to reduce cardiac dose (Wang *et al* 2012). The device has demonstrated a good inter-fraction reproducibility of breath holding for CT scans (Sarrut *et al* 2005). Nevertheless, to our knowledge it has never before been applied during MRI studies.

Eichinger *et al* (2007) have already performed MR-compatible spirometry but without arresting breathing, by modifying a different commercial system built by Viasys Healthcare which employs a Lilly pneumotachograph to record air pressure changes. Arnold *et al* (2007) also used a modified Viasys spirometer with an additional shutter device capable of inducing breath-holds. They demonstrated improvements in electrocardiography (ECG)-triggered MRI under enforced breath holding compared to free breathing and to gating using a respiration belt. Halaweish and Charles (2014) acquired conventional, fluorine-enhanced, oxygen-enhanced and Fourier decomposition free breathing and breath held MR images using Biopac pneumotachometers. They state that breathing manoeuvres are repeatable, yet they are accomplished through verbal cues and therefore depend on subjects’ compliance and operator reaction.

As pneumotachographs measure gas flow by detecting pressure differences, they are very sensitive to temperature, humidity and atmospheric pressure of surrounding air (Cotes *et al* 2009) and need to be calibrated very often. In contrast, turbine spirometers such as the ABC provide reliable and reproducible results and need no frequent calibration. Additionally, the ABC allows breath hold enforcement when a specified volume threshold is surpassed, thus achieving repeatable lung volume between different breath holds. This feature is very useful for longer MR sequences, which need to be acquired in several breath holding segments. Given the wide use of the ABC during lung radiotherapy (McNair *et al* 2009) and the possibility of fusing MR with CT images, we aimed to apply such a respiratory control apparatus during MR scanning with the least possible modifications and the highest degree of automation. Our ultimate goal was to acquire MR images with the same patient positioning and lung volume as during treatment planning and radiotherapy, to improve the planning and to aid the assessment of treatment response.

To this end, we have successfully modified and tested an ABC device for MR application at 1.5 T, optimized examination protocols for MR-ABC use and demonstrated its feasibility for morphological and functional MRI on healthy volunteers and five patients. We assessed the advantages of MR-ABC, the consistency of ABC-controlled breath holding within a MRI session and between the MR and CT examinations, as well as the diagnostic potential of additional information obtained by MR imaging in ABC breath holds with the same settings as during CT.

## 2. Methods

### 2.1. ABC description

The working principle of the ABC is the following (Elekta Limited 2012): an individual with a clamped nose breathes through an extendable 20–44 cm long breathing tube via a mouthpiece equipped with a filter at the distal end. The resulting air flow turns the impeller in the inline turbine cartridge of the attached digital volume transducer. The direction and number of impeller rotations are detected by opto-electrical detectors within the transducer’s pick-up assembly. The electrical signal produced is transmitted to a control module where it is converted and displayed as a volume curve on a computer executing the control software. The operator presets a volume threshold for breath holding on in- or exhalation and the breath hold duration in the control software. To prepare a breath hold, the operator activates the system by pressing the space bar on the control computer and instructs the individual to breathe deeply in or out. When the air volume exceeds the preset inhalational or exhalational volume threshold, respiration arrest is automatically achieved for the predefined duration by inflating a balloon valve to occlude the airway. Patient compliance and safety are guaranteed by a handheld electrical switch, which deflates the balloon valve when released. The operator can also terminate a breath hold early from the control computer. As an additional safety aspect, the mouthpiece can be easily ejected by subjects in case of emergency.

### 2.2. ABC volumetric assessments

In order to confirm the readings of the ABC control box, inspect possible effects of airflow on recorded volume and assess the ABC volume transducer performance outside a scanner’s magnetic field, bench tests were performed. Signals from the ABC volume transducer were independently assessed by a custom-made circuit using a National Instruments (NI) data acquisition (DAQ) card controlled by LabVIEW (National Instruments, Austin, Texas, USA), during manual delivery of fixed air aliquots using a 3L calibration syringe (Hans Rudolph Model 5530) and analysed offline using MATLAB (Mathworks, Natick, MA).

### 2.3. MR compatibility assessments

Tests of the MR compatibility of the ABC patient respiratory system, consisting of breathing tube with filter, digital volume transducer, balloon valve and couplers, were performed in a 1.5 T scanner (Magnetom Avanto; Siemens, Erlangen, Germany). Observations were confirmed for another 1.5 T scanner (Magnetom Aera; Siemens, Erlangen, Germany). The respiratory system does not pose a projectile risk, even though the pick-up assembly contains small metallic parts and is very slightly magnetic.

To inspect if and how the ABC presence and function affects MR images, axial three-dimensional Fast Low Angle Shot (3D FLASH) scans of a cylindrical flood phantom were performed with parameters reported in table 1. A very low flip angle was used to increase the noise sensitivity of the sequence. At first the ABC respiratory system was not present in the scanner room, then it was taped on the phantom side at the scanner isocentre plane and lastly placed at an axial distance 30 cm from the isocentre. During the last two conditions a person was breathing heavily in the ABC system and the transducer cable was connected to the electronic readout devices outside the scanner room with custom connectors through the radiofrequency (RF) panel. For all three conditions, images were also acquired at 60 kHz off resonance for noise assessments. Signal to noise ratio (SNR) was calculated for each slice as the mean intensity in a phantom circular region of interest (ROI) on the on-resonance image divided by the standard deviation of the intensity values in the same ROI on the off-resonance image.

**Table 1.** Parameters of the phantom and volunteer measurements.

	3D FLASH	DW-EPI
Number of slices	16	33
Slice thickness (mm)	5	6
TR (ms)	11.7	1100
TE (ms)	4.76	67
FoV (mm^2^)	379 × 379	308 × 380
Matrix size	192 × 192	208 × 256
Flip angle (°)	2	90
Bandwidth (Hz/px)	200	1776
Acceleration factor	None	GRAPPA 2
*b*-values (s mm^−2^)		0, 100, 500, 750

Assessments of potential impacts of the MR scanner on the ABC readings were accomplished by repeating the bench experiments using the 3L calibration syringe with the ABC transducer placed at the scanner isocentre, either parallel or perpendicular to the static magnetic field *B*_0_. These ABC volume readings were compared to the bench results.

### 2.4. Modifications for MRI use

**Figure 1.**
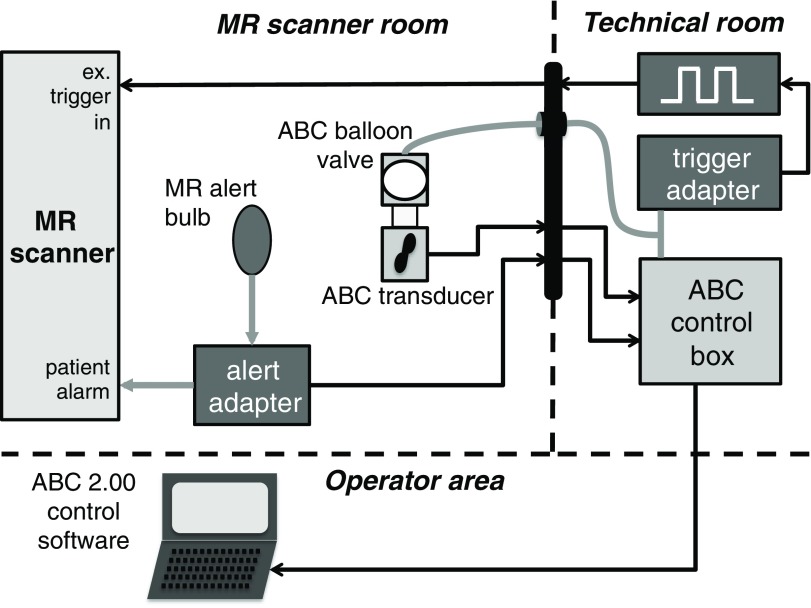
Schematic MR-ABC diagram: the original ABC digital volume transducer in the MR scanner room is connected through the RF panel to the original ABC control module in the technical room using custom extension cables and connectors. The tubing of the original balloon valve is extended to reach the ABC control box and the purpose-built trigger adapter. Upon detection of full balloon valve inflation, the adapter activates a waveform generator that triggers MR acquisition. Patient alert signals from the MR patient alert button are intercepted by a custom adapter and fed into the patient switch input of the control module to abort breath holding. The module communicates with a control computer in the MR operator area, operating the standard ABC control software. Grey lines indicate air tubes while black lines denote electrical connections. Boxes with a darker hue represent components different from the standard ABC design.

A schematic diagram of the MR-ABC apparatus and connections, applicable for both 1.5 T scanners used, is shown in figure 1.

#### 2.4.1. Equipment placement adjustments.

The control module was located in the scanner’s technical room; it has to remain outside the MR scanner room to avoid RF interference and interactions with the magnetic field. Consequently the volume transducer and balloon valve are further from the control module than the standard distance for CT scanning or radiotherapy treatment and their signals must pass through the scanner room RF screen. For this purpose, RF-filtered panel adapters were fitted to the RF panel for each scanner. The original vendor’s cable for the volume transducer was extended with custom cables via the filtered panel adapter.

The standard 2.25 m long ABC balloon valve tubing also needed to be elongated, by either 3.05 or 4.60 m for use in the 1.5 T Siemens Aera or Avanto respectively, in order to reach the ABC control module. The balloon valve inflation and deflation delay was experimentally observed to increase linearly with tube length and amounted to 0.7 s, 1.4 s and 1.7 s for the standard, Aera and Avanto ABC applications respectively. These values include intrinsic delays of the control module, which were estimated in laboratory testing to be 0.18 s. Since the balloon inflation delay reduces the time the valve is fully closed, the breath hold duration set at the ABC software prior to MR scanning was increased by the inflation delay for the applied tube length to guarantee complete valve closure for the desired time span.

The ABC control computer was located in the scanner operator room connected directly to the control module by a serial link, without using the standard extender modules, and ran the standard Elekta ABC control software version 2.00.

#### 2.4.2. Patient switch substitution.

The original ABC patient control switch is an electrical single pole (on-off switch). It is intended to be pressed closed continuously by the patient throughout an ABC session to indicate compliance and enable breath control. If the switch opens it is not possible to inflate the balloon valve or an already inflated balloon becomes deflated. On the other hand, a standard safety feature of MR scanners is a pneumatic patient alert bulb that is squeezed to provide an alarm signal. In order to avoid patients having to operate two hand controls with opposite operating modes, we modified the MR alert system by connecting an electro-pneumatic switch into the pneumatic line. The normally closed contacts on the pressure switch are connected to the patient switch input of the ABC control module via the RF panel adapter. If the alert bulb is pressed the contacts open and balloon valve inflation is suspended. The sense of the switch is unavoidably reversed between MR scanning and normal ABC operation but in either environment changing the state of the switch during a breath hold causes the ABC valve to open. The patient alert signal to the scanner is not affected by this modification.

#### 2.4.3. Additional scanner triggering.

In order to achieve automatic and simultaneous MR acquisition with ABC-induced breath holds, a custom circuit was built to detect full balloon valve closure and to externally trigger MR sequences during its duration. The device measures the balloon valve pressure and provides a signal to the external triggering input on the scanner. The trigger frequency required by the scanner varies according to the particular sequence of a patient examination protocol. The circuit either output directly a particular triggering frequency during the early volunteer scans or, as displayed in figure 1), triggered an Agilent 33250A 80 MHz Waveform Generator (Agilent Technologies Inc., Loveland, Colorado, USA) to provide an accurate stream of trigger pulses at an easily specified rate.

#### 2.4.4. Patient and spirometer positioning.

**Figure 2.**
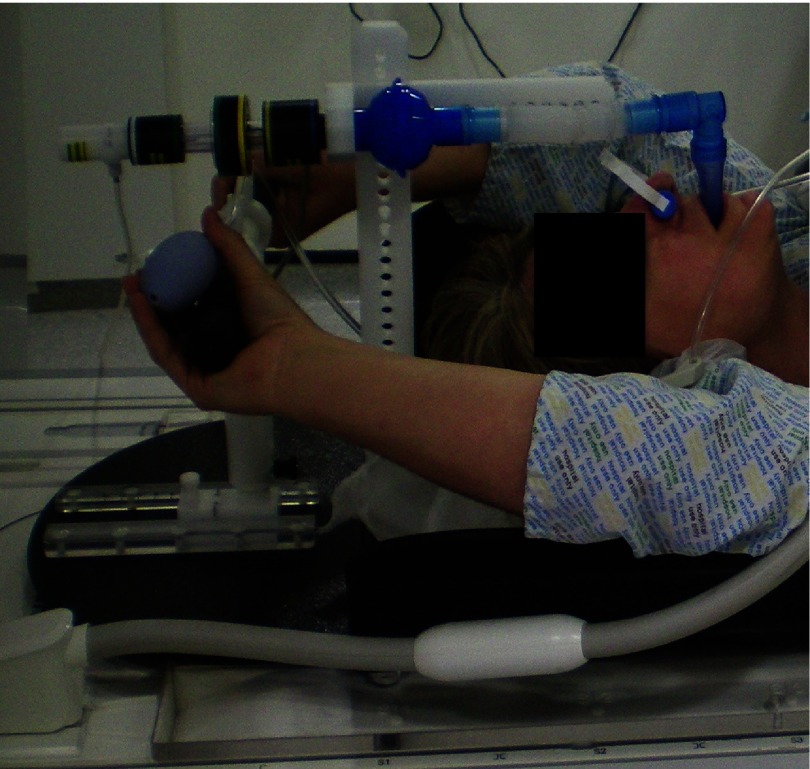
Patient setup for MR-ABC examinations: subjects lay supine on an Extended Wing Board fixed on a Perspex flatbed, with hands superior to their head and their nose clamped. The ABC breathing system, spirometer and balloon valve were supported by an adjustable purpose-built post. A modified MR alert button replaced the original patient switch and was retained in position by custom clamps on the handle bar.

To ensure that patient positioning was as similar as possible between MR and CT examinations and radiotherapy delivery, an identical patient positioning board was used (Extended Wing Board, Oncology Systems Limited, Shropshire, UK). Such a board is depicted in figure 2 and features a headrest, two ridges to support the elbows and an adjustable post with a handle bar. The standard radiotherapy metallic support post of the ABC respiratory system fixed on the Wing Board was substituted by a purpose-built MR compatible one. The custom pole held the ABC spirometer and balloon valve parallel to *B*_0_ at an adjustable height and distance from the pole. A right angle connector was used between mouthpiece and breathing tube, which was kept to its shortest length to minimize dead space. Custom clamps were attached on the handle bar to ensure that the modified MR alert button remained in the subject’s hand. The modified Wing Board was fixed on a radiotherapy planning Perspex flatbed placed on the scanner couch for patient examinations.

### 2.5. Human imaging

Various T1, T2, T2* and DW MR sequences, a subset of which is mentioned in table 2, have been successfully applied and optimized during volunteer studies with the modified ABC system in a 1.5 T Siemens Avanto and 1.5 T Siemens Aera. Clinical evaluations of the MR compatible ABC were performed in the Aera by reason of its wider bore (60 cm). MR-ABC studies were approved by the Institutional Review Board and the participating volunteers and patients were consented. All persons who underwent an MR-ABC examination tolerated it very well.

**Table 2.** Parameters of the MR-ABC patient examination protocol.

	VIBE	HASTE	DW-EPI1	DW-EPI2
Number of breath holds	1	4	2	3
Breath hold duration (s)	17	16	17	16
Number of slices	80	60	30	30
Slice thickness (mm)	3	3	5	6
TR (ms)	4	1000	3300	4000
TE (ms)	0.94	100	45	62
FoV (mm^2^)	270 × 360	261 × 380	258 × 376	259 × 377
Matrix size	144 × 256	132 × 256	192 × 280	88 × 128
Flip angle (°)	8	170	90	90
Acceleration factor	GRAPPA 2	GRAPPA 2	GRAPPA 2	GRAPPA 2
*b*-values (s mm^−2^)			200	100, 400, 750
Number of averages	1	1	2	1

#### 2.5.1. MR-ABC examination procedure.

Subjects were at first acquainted with the MR-ABC device while sitting on a chair in the scanner room. The proper function of the system, subject compliance with the breath holding requirements and safety aspects were checked by monitoring subject respiratory volume curves, inducing a few breath holds and testing the modified MR alert button. During the MRI session, subjects were lying supine with their nose clamped and the mouthpiece air tightly fitted in their mouth. To ensure their welfare they were video surveyed and their breathing patterns were observed by the operators. To perform a breath hold, the operator armed the ABC and instructed subjects to take a deep breath when ready. When the inhaled air volume exceeded the threshold predefined for the particular subject, the balloon valve closed and MR acquisition was automatically externally triggered for as long as the valve remained shut. The pre-set breath hold duration was determined by combining subjects’ respiratory capabilities with the possible segmentations of the total MRI acquisition time, and took into account the balloon valve inflation delay. The remaining breath hold time was counted down to the subject, in seconds, via the intercom device. Upon valve reopening MR scanning stopped automatically and subjects were instructed to breathe normally. In case a sequence was divided into multiple segments the above procedure was repeated accordingly. If a MRI sequence would not support external triggering, the operator would instruct subjects and initiate a breath hold as above but would manually start the MRI acquisition when the monitored volume curve reached its specified plateau.

#### 2.5.2. Volunteer studies.

From the volunteer studies, we describe here abdominal DW echo-planar imaging (EPI) of a young healthy male lying supine in the Avanto with arms superior to his head. The ABC breathing system, spirometer and balloon valve were fitted on him and axial images were acquired with parameters shown in table 1, applying spectral adiabatic inversion recovery (SPAIR). At first, the EPI sequence of nominal duration 73 s was implemented in five volunteer-sustained breath holding segments at inhalation, under the common practice of operator guidance. Afterwards the same sequence was repeated twice, acquired in segments defined and externally triggered by ABC-induced breath holds at 1.2 L inhaled air volume for a pre-set span of up to 15 s.

Images from the two ABC-controlled measurements were visually compared, averaged and subtracted at the same slice locations to evaluate intra-session organ position reproducibility under ABC control. The quality of apparent diffusion coefficient (ADC) maps produced by aggregating images from these two measurements was also assessed. Moreover, images from all three acquisitions were reformatted and compared in coronal orientation. In order to evaluate ABC performance over all slices, coronal image subtraction of the last two measurements at the same slice location was performed too.

#### 2.5.3. Patient studies.

Five lung cancer patients underwent contrast-enhanced CT scanning (Brilliance Big Bore, Philips Healthcare, DA Best, The Netherlands) using 2 mm slice thickness and spacing, in one 20 s long ABC-controlled breath hold and in the treatment position on an Extended Wing Board. The breath hold volume was individually defined for each patient at 70% of his or her maximum inspiratory volume. About two weeks later these patients were scanned in a 1.5 T Siemens Aera, lying supine on the modified Extended Wing Board and the flatbed with arms superior to their head, as displayed in figure 2. The position and height of the handle bar were matched to the CT settings. Radiotherapy tattoos were aligned by using sagittal and axial lasers fixed on the MR scanner walls.

Morphological and functional MR sequences were acquired in ABC breath holds with the same individual inhalational volume threshold as during CT. In particular, 3D T1-weighted volume interpolated breath-hold examination (VIBE) images were obtained during a single ABC-induced breath hold. A T2-weighted half Fourier acquisition single shot turbo spin echo (HASTE) and two DW-EPI sequences were acquired in segments externally triggered by ABC-controlled breath holds. Detailed sequence parameters are listed in table 2. The whole patient examination protocol was completed in about 20 min.

For tumour and thorax position intra-session reproducibility assessments, images from the different MR sequences of the same patient and session at similar slice positions were visually compared and overlaid in colour scale using the OsiriX software (Rosset *et al* 2004) without applying any registration. Registration between CT and morphological as well as functional MR images was evaluated by automatically fusing them applying a local correlation algorithm in the radiation treatment planning software Pinnacle^3^ (Philips Radiation Oncology Systems, Fitchburg, WI).

## 3. Results

### 3.1. Phantom tests

**Figure 3.**
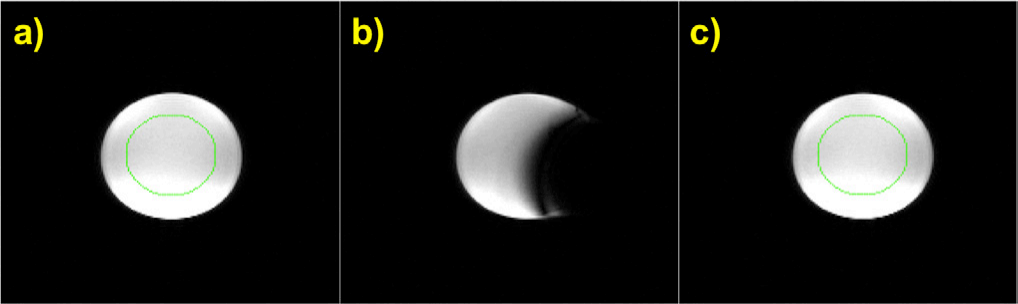
The same axial slice with (*a*) no ABC in the scanner room, (*b*) ABC transducer at the scanner isocentre plane and (*c*) ABC transducer 30 cm far from the isocentre in axial direction. All images are equally windowed. The circular ROI used for SNR calculations is drawn on (*a*) and (*c*).

Figure 3 displays the same cylindrical flood phantom slice for the three conditions tested. Taping the ABC respiratory system on the phantom side so that the ABC transducer lies on the isocentre plane causes signal cancellation due to susceptibility artefacts arising from the transducer’s metallic parts (*b*). However, images with the ABC placed 30 cm further from the isocentre towards the scanner rear (*c*) do not differ from images with no ABC at all (*a*). SNR assessments revealed that the mean SNR difference between all 16 slices without versus with the ABC at 30 cm is (−2  ±  8)% (mean ± standard deviation). The mean SNR of the 7 median slices was (196  ±  10) arbitrary units (a.u.) without and (192  ±  10) a.u. with the ABC at 30 cm. These results demonstrate that MR image quality is not affected by the presence and function of the ABC respiratory system at a distance ≥ 30 cm from the scanner isocentre.

Compared to the bench data, the recorded ABC volume was not affected if the transducer was oriented parallel to *B*_0_. It was uniformly reduced by about 1.5% of the nominal air volume if the transducer was perpendicular to *B*_0_ due to eddy current braking on the rotating impeller’s metallic shaft. Taking these findings into account, the transducer’s position was always fixed parallel to *B*_0_ and at least 30 cm from the scanner isocentre for MR-ABC applications.

### 3.2. Volunteer studies

**Figure 4.**
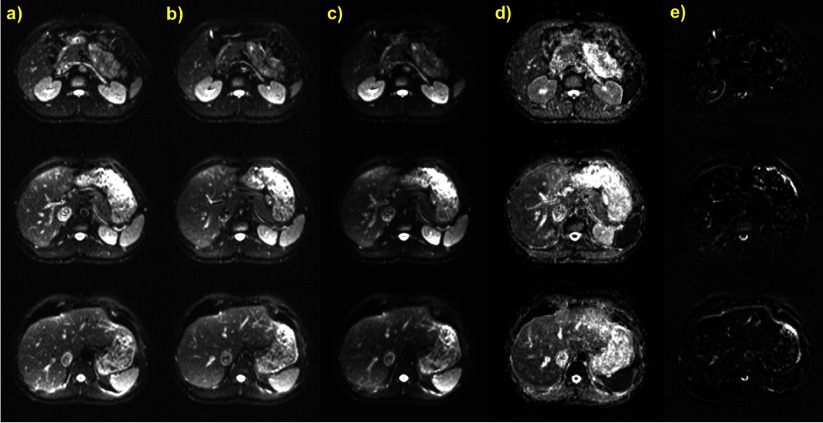
Three abdominal slices of a DW-EPI of a healthy volunteer acquired during multiple ABC breath holding with the same settings at two time points (*a*) and (*b*) during the same session (*b* = 0 s mm^−2^, identical slice position) to demonstrate averaging with ABC breath holds. (*c*) is the average of (*a*) and (*b*). (*d*) ADC maps produced by aggregating the DW images from the time points (*a*) and (*b*) at each slice position (*b* = 0, 100, 500, 750 s mm^−2^). (*e*) Difference images (*b*) − (*a*). No image registration was used in any operation and displayed windowing is the same in all cases except for the ADC maps.

Three sample slices of the volunteer measurement containing the liver are shown in figure 4 for *b* = 0 s mm^−2^. The position of organs such as the liver, kidneys, pancreas, stomach and spleen is obviously well replicated in the two different segmented acquisitions (*a*) and (*b*) in breath holds induced by the ABC at the same lung volume threshold. It is therefore possible to average images (*a*) and (*b*) as in (*c*) without applying any kind of image registration and obtain an increased SNR, e.g. by around 40% in the liver. ADC maps in (*d*) produced by aggregating (*a*) and (*b*) using all *b*-values demonstrate a detailed distinction of abdominal structures, e.g. differentiation of the renal cortex and medulla, and minimal boundary effects. The difference images (*b*) − (*a*) in (*e*), again without any coregistration, also suggest a very good repeatability of organ position. Residuals are associated with blood flow, peristalsis and ghosting artefacts in EPI sequences.

**Figure 5.**
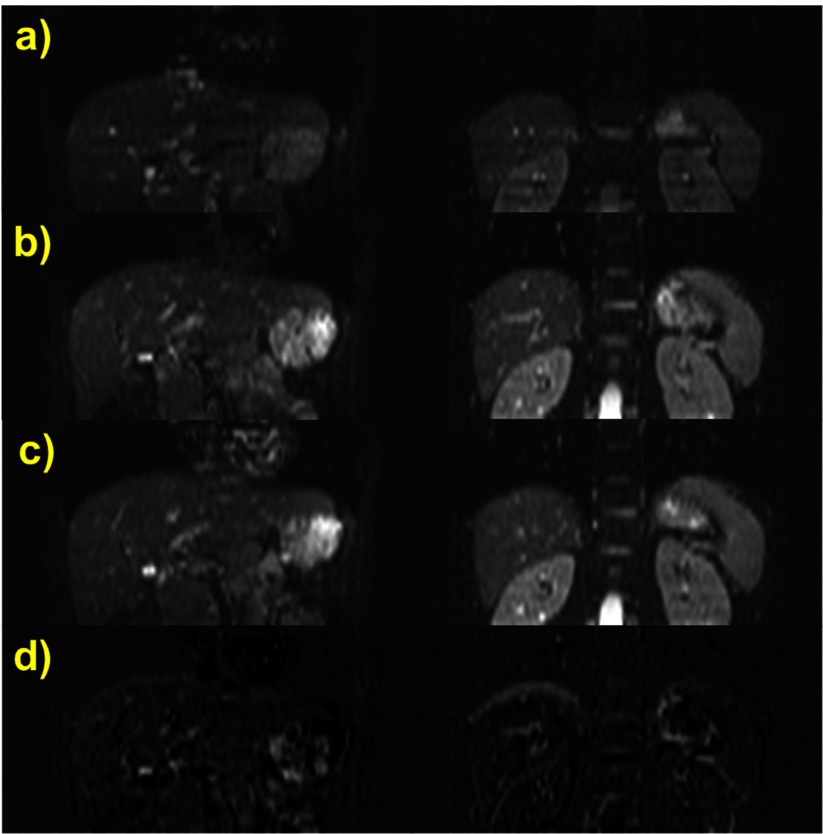
Two coronally reformatted slices of a DW-EPI (*b* = 0 s mm^−2^) of a healthy volunteer acquired during (*a*) standard operator-instructed self-induced multiple breath holds; (*b*) ABC-controlled multiple breath holding and (*c*) repetition of (*b*). (*d*) Difference images (*b*) − (*c*), demonstrating good intra-session registration using the ABC. No image registration was used and the slice location and windowing is identical for all displayed images.

Two coronally reformatted slices of the standard operator-guided multiple breath holding technique are depicted in figure 5(*a*) for *b* = 0 s mm^−2^. They show dark horizontal bands, presumably caused by motion or abdominal organ position variations during the self-sustained breath holds. The coronal reformatting of the two ABC-controlled data sets (*b*) and (*c*) at the same slice location shows better image quality and minimisation of such artefacts. The liver, kidney, stomach and pancreas position is very similar in (*b*) and (*c*) but shifted in (*a*), suggesting that ABC-induced breath holds were performed at the same but self-sustained ones at a different inhalational level. Subtracting the ABC images in (*d*) indicates good agreement between repeated acquisitions under ABC control and reveals minor variations due to blood flow, peristalsis and ghosting. As the diaphragm is a very deformable organ, slight differences in its position are not surprising and have been observed in the past during self-induced breath holds (Holland *et al* 1998)

### 3.3. Patient studies

**Figure 6.**
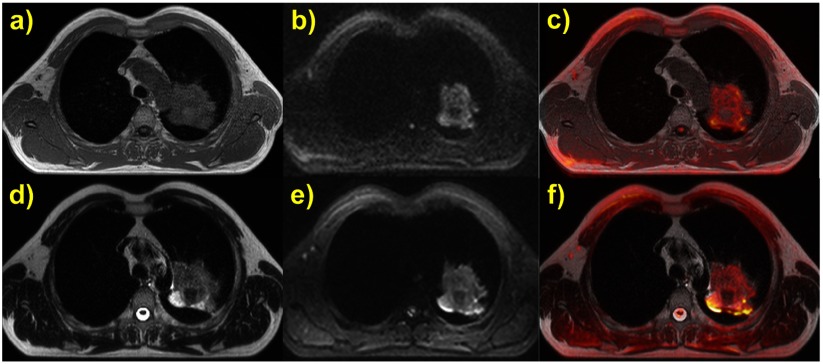
MR images of a NSCLC patient acquired in ABC breath holds with the same inhalational threshold: (*a*) T1-w 3D VIBE (slice location 57.56 mm, thickness 3 mm); (*b*) DW-EPI2 with *b* = 750 s mm^−2^ (slice location 57.17 mm, thickness 6 mm); (*c*) overlay of (*a*) and (*b*) in OsiriX. (*d*) T2-w HASTE (slice location 58.67 mm, thickness 3 mm); (*e*) DW-EPI1 with *b* = 200 s mm^−2^ (slice location 58.67 mm, thickness 5 mm) and (*f*) their overlay in OsiriX. All original images were individually windowed.

Example morphological and functional MR images of a non small cell lung carcinoma (NSCLC) patient acquired during ABC breath holds at 2.6 L inhaled air volume are displayed in figure 6 at a similar slice position featuring the tumour. Overlaying a *b* = 750 s mm^−2^ DW-EPI2 on an anatomical T1-w VIBE by using OsiriX without any image registration (figure 6(*c*)) showed a very good agreement on tumour and spinal cord position and a fairly good replication of the thorax contours, taking into account the different slice thickness and EPI distortions in the anterior to posterior (A–P) phase-encoding direction which are expected to be quite strong for such a relatively high *b*-value (Koh *et al* 2007). Likewise, overlaying the higher spatial resolution DW-EPI1 with *b* = 200 s mm^−2^ on the T2-w HASTE resulted in very good matching of the tumour and thoracic structures, considering the difference in slice thickness.

The T1 and T2 weighted images (*a*) and (*d*) also seem to agree on tumour size, despite their slice location differing by 1 mm. The HASTE and EPI acquisitions demonstrate intensity variations in different parts of the tumour, possibly indicating areas with differences in fluid content and diffusion properties. The applied combination of T1, T2, and DW sequences with different *b*-values and resolution in the MR-ABC patient examination protocol can be useful to identify and exclude possible confounding imaging effects, to discriminate different pathological tissue areas and subsequently to improve and verify diagnostic predications.

**Figure 7.**
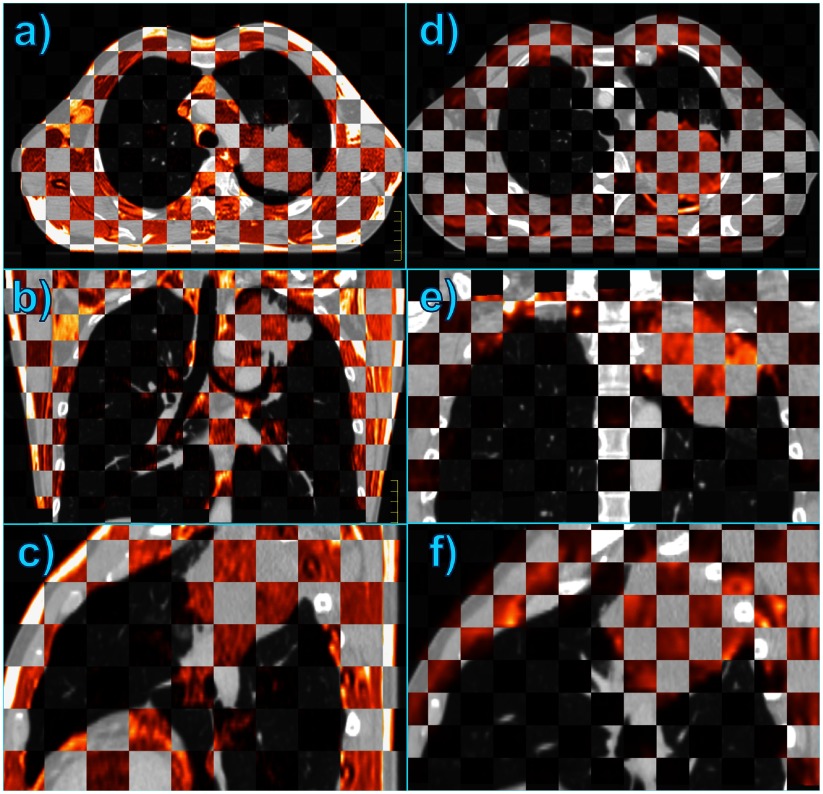
(*a*) Checkerboard view of the Pinnacle^3^ registration of an axial CT image (grey) with an axial 3D T1-w VIBE image (orange) of a NSCLC patient in ABC-controlled breath holding with the same settings and positioning. (*b*) Coronal and (*c*) sagittal reconstruction of the CT-VIBE fusion. (*d*) A more superior transversal slice of the CT image of the same patient (grey) fused with a *b* = 200 s mm^−2^ DW-EPI1 (orange) acquired under the same ABC conditions. (*e*) and (*f*) denote the coronal and sagittal reconstruction of the CT-EPI fusion. All of these images demonstrate the good inter-modality registration achievable with the ABC system.

Example results from the fusion in Pinnacle^3^ of CT images of a NSCLC patient (grey), with MR images (orange) acquired 19 days later in ABC-controlled breath holding at the same lung volume and patient positioning, are shown in figure 7 in a checkerboard view. This display is used in radiotherapy treatment planning to evaluate image fusion quality by comparing the position of anatomical features in adjacent squares corresponding to two different image sets (Wang *et al* 2009) The registration of the 3D anatomical T1-weighted VIBE with the CT image appears very similar, not only on the acquired axial orientation (*a*) but also for the reconstruction in the other two directions (*b*) and (*c*). The lung and tumour borders, bony structures such as the ribs and spinal cord, the skin, even the aorta and major vessels coincide very well on the acquisitions from the two different modalities.

The fusion of the CT with the *b* = 200 s mm^−2^ DW-EPI1 image, as displayed for a more apical slice in (*d*), yields a very satisfactory agreement too, considering potential EPI distortions. The coronal and sagittal reconstruction (*e*) and (*f*) also show a good match of the inner and outer thorax and of the pathology delineation, despite the 2D character of the applied EPI sequence. DWI images present more heterogeneous intensity patterns on the tumour than CT, which may reflect structural and biological differences inside the cancerous mass. A higher intensity band visible on the original transversal EPI reveals fluid accumulation in the pleural cavity. The DW MR acquisition distinguishes the tumour boundaries from the pleural effusion whereas the CT acquisition shows just a homogenous intensity area.

## 4. Discussion

### 4.1. Modifications for MR use

A commercial Active Breathing Coordinator system was successfully modified for MR use by adding, extending or replacing peripheral components but without altering its digital volume transducer or main control unit. The ABC spirometric readings were verified by independent custom electronics and software. Investigations showed that the presence and function of the transducer might compromise MR images if placed at the isocentre of a 1.5 T magnet, but does not affect them at 30 cm axial distance from it. As the total length from the proximal end of the breathing tube to the centre of the pickup amounts to at least 31 cm for a completely contracted tube, thoracic or abdominal imaging of even the smallest individuals is feasible without any ABC transducer artefacts. Care was taken to fix the transducer oriented parallel to *B*_0_, to avoid slight reductions in the displayed volume caused by eddy current braking at different angles.

Electric cable extensions and custom connectors did not affect the ABC performance or fidelity of the MR scanner Faraday cage. However the pneumatic tube elongation required for operation in an MR scanner increased the time needed to fully open and close the balloon valve. We anticipate that planned modifications to the airway valve will significantly reduce its reaction time. Nonetheless respiration arrest could be achieved for the desired time with the present configuration, since the overall balloon valve inflation delay for the total applied tube length had been measured and included in the breath hold duration on the ABC software. The custom-made trigger circuit detected full valve closure and externally triggered MR scanning from the onset until the termination of an ABC-controlled breath hold. The automatic MR acquisition during actual respiration arrest achieved with this innovation was an improvement of the original system, which relies on the operators to manually initiate imaging or irradiation and may be compromised by their reaction time.

The modification of the MR alert button to additionally act as an ABC patient switch reversed the original arrangement of releasing the switch in case of respiratory discomfort. Since the alert button is a safety requirement for MR scanning and to avoid patient confusion with two differently functioning switches, we preferred to retain the MR bulb only and follow the ordinary habit of pressing it in an emergency. The custom patient switch adapter causes the ABC to open the airway valve when the MR bulb is squeezed. The standard ABC control software was used to ensure conformance with CT examinations and radiotherapy treatments. An identical patient positioning board with a custom-built spirometer support and the use of radiotherapy tattoo laser alignment allowed for replication of patient position between CT and MR examinations.

### 4.2. General MR-ABC applicability

Altogether, minimal alterations to the standard ABC commercial system were performed to use it in two 1.5 T Siemens scanners. The proposed MR-ABC design could be well reproduced for other scanner types, albeit probably with minor technical adjustments. External triggering is more a desirable feature than a necessity, and its practicability should be investigated and optimized for the intended scanners, clinical applications and sequences. ECG triggering would also be feasible during MR-ABC scanning to additionally suppress cardiac motion artefacts but was avoided in this work in order to maintain a short total breath holding time, especially considering lung cancer patients’ physical limitations. The number and duration of sequence segments can be selected according to the breath holding capabilities of the subject. MR image quality and the proper function of the original ABC digital volume transducer should be tested for application at higher field strengths. Modifications of the standard transducer might improve its MR compatibility.

### 4.3. MR-ABC outcome

The main incentive for modifying the Elekta ABC device for MR application instead of following other motion compensation techniques (van Heeswijk *et al* 2012) or already developed MR-compatible breathing control systems based on pneumotachographs (Arnold *et al* 2007, Eichinger *et al* 2007, Halaweish and Charles, 2014), was that the commercial device is widely used in lung radiotherapy. As recent advances in morphological and functional MRI allow for detection, characterization and staging of pulmonary nodules with a diagnostic quality equivalent to that of CT and positron emission tomography (PET)-CT (Hochhegger *et al* 2011), and given the ABC’s ability to arrest respiration at an individually defined and reliably reproducible inhaled or exhaled air volume, we anticipated that MR and CT images would match for the same ABC settings and patient positioning.

The MR-ABC system was first tested on healthy volunteers and the assumption that the lung volume remains constant between breath holds with the same inhalational or exhalational threshold was validated. Comparing ABC-induced to self-sustained segmented T2-weighted selective single slab 3D Sampling Perfection with Application optimized Contrasts using different flip angle Evolution (SPACE) images has already revealed improved thoracic organ delineation for ABC-controlled breath holding (Kaza 2013), which reflects better lung volume reproducibility in the ABC case. As described in the present work, repeating a DW-EPI sequence acquired in breath holds managed by the ABC device in the same scanning session delivered better image quality than self-sustained breath holding and showed very good agreement between corresponding slices and precise abdominal organ position reproducibility. Consequently, images from the two acquisitions could be averaged to increase SNR and improve ADC maps.

The MR sequence protocol was optimized for patient examinations under ABC control in order to keep acquisition time as short as possible while including different kinds of contrast weighting to maximize diagnostic potential. Using the protocol described, valuable T1, T2 and DW images could be obtained in a total of 10 breath holds of 16 or 17 s duration each that all five examined patients managed very well. In order to assess MR and CT image registration care was taken to reproduce patient positioning between the two sessions. MR images showed not only a very good intra-session tumour and thorax position reproducibility, but also matched very well with CT images acquired 2–3 weeks earlier. A combination of variously weighted MR images could distinguish tumour areas with different composition and content, as well as provide additional information about pleural effusion, which was not discernible on CT images.

### 4.4. Further MR-ABC potential applications

In the scope of this work we demonstrated that the proposed MR-ABC system improved lung imaging in breath hold. The device is furthermore expected to be equally applicable in abdominal examinations aiming at eliminating respiratory motion, for instance to image liver or pancreas pathologies. Oesophageal imaging should be possible too, preferably combined with simultaneous ECG triggering to suppress cardiac motion artefacts. Once an MR protocol is established for a particular examination under ABC control, its results may serve as a Gold standard for the development of new acquisition protocols. Moreover, MR-ABC can provide spirometric data during synchronized dynamic MRI, which would be very useful for motion studies of healthy tissues or of pathology. It offers the possibility to track a specific respiratory phase, which may be useful for gating purposes such as triggering an MR-linac.

## 5. Conclusion

A commercial Active Breathing Coordinator device was successfully modified for MR application for the first time and its feasibility was demonstrated on healthy volunteers and five lung cancer patients. A very good intra-session reproducibility of organ position and improved image quality was demonstrated under MR-ABC control. MR-CT registration appeared very similar for the same ABC settings. The acquired MR images provided additional diagnostic information, which will be essential for improved radiotherapy treatment planning and assessment.
